# The Hypometabolic State of the Migraine Brain: Is a Ketogenic Diet the Answer?

**DOI:** 10.1002/brb3.70860

**Published:** 2025-09-10

**Authors:** Lakshini Gunasekera, Jason C. Ray, Helmut Butzkueven, Terence J. O'Brien, Elspeth Hutton, Neha Kaul

**Affiliations:** ^1^ Department of Neuroscience, School of Translational Medicine Monash University Melbourne Australia; ^2^ Department of Neurology Alfred Health Melbourne Australia; ^3^ Department of Neurology Austin Health Melbourne Australia; ^4^ Department of Nutrition and Dietetics Alfred Health Melbourne Australia

**Keywords:** ketogenic diet, migraine, metabolism

## Abstract

**Background:**

Migraine pathophysiology involves a constellation of metabolic abnormalities. These interlinked contributory factors include mitochondrial dysfunction, an altered gut microbiome, neuroinflammation, oxidative stress, weight imbalance, and altered glucose metabolism. The ketogenic diet is an emerging therapy which may restore hypometabolism seen in chronic migraine. We describe contributions of metabolic dysfunction to chronic migraine pathophysiology and discuss the role of ketogenic diet therapy to improve cerebral metabolism.

**Methods:**

A literature search of articles from OVID Medline, Embase, and Cochrane Library were reviewed until May 6, 2024. A total of 50 articles were included comprising case reports, case series, observational clinical trials, and narrative review articles.

**Results:**

Migraine pathophysiology involves significant hypometabolism, seen on functional imaging studies, so therapeutics which target these underlying deficiencies may ameliorate chronic migraine symptoms. The ketogenic diet reduces migraine days, pain intensity, and acute analgesic use. Current studies are limited by small sample sizes, inconsistent methodology precluding direct comparison between studies or pooling of results, and limited longitudinal follow‐up.

**Conclusion:**

While there is biological plausibility to reason that a ketogenic diet could correct metabolic mismatch in people with migraine, further randomized clinical trials with larger sample sizes are required to confirm the positive results of preliminary, uncontrolled studies.

## Graphical abstract

The pathophysiology of migraine attacks involve metabolic factors such as altered gut microbiome, sterile neurogenic inflammation, cortical spreading depression, mitochondrial dysfunction, obesity, and altered glucose metabolism. Preliminary studies show that a ketogenic diet may reduce cerebral metabolic mismatch through these mechanisms to cause symptomatic relief in patients with migraine.

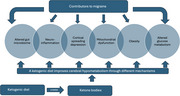

**Abbreviations**
BMI: body mass index
CM: chronic migraine
HBD: hypocaloric balanced diet
HFEM: high frequency episodic migraine
HIT6: Headache Impact Test
KD: ketogenic diet
LCD: low‐calorie diet
LGID: low glycemic index diet
MIDAS: Migraine Disability Assessment
MMD: monthly migraine days
SD: standard diet
VLCKD: very low calorie ketogenic diet
VLCnKD: very low calorie non ketogenic diet

## 1 Introduction

The role of metabolic dysfunction in migraine was first described in 1935 when it was recognized that hypoglycemia provoked migraine, and that its correction led to headache resolution (Gray and Burtness 1935). Later, in 1982, lactate was shown to be elevated in serum and cerebrospinal fluid of people with migraine during an attack, leading to the hypothesis that cerebral hypoxia was also contributory to migraine (Amery 1982). Taken together, both these early hypotheses support likely underlying metabolic dysfunction as a component of migraine pathophysiology.

Our current understanding of migraine pathophysiology is that these headaches are, in at least part, due to a constellation of metabolic abnormalities (Gazerani 2021). These interlinked contributory factors include mitochondrial dysfunction (di Lorenzo et al. 2021; Lai and Niddam 2020; Wang et al. 2023; Gross et al. 2021), an altered gut microbiome (Finkel et al. 2013; Chen et al. 2019), neuroinflammation and oxidative stress (Gross, Klement, et al. 2019; Sun‐Edelstein and Mauskop 2009; Finelli et al. 2024), weight imbalance (Rainero et al. 2018; Bigal et al. 2006; Vincenzo et al. 2020; Martin and Vij 2016), and altered glucose metabolism (Gross, Klement, et al. 2019; Rainero et al. 2018; Hassan et al. 2020; Del Moro et al. 2022). There is emerging evidence that a ketogenic diet (KD) may restore cerebral hypometabolism, by correcting these interlinked contributory factors, to alleviate headache burden (Schnabel 1928; Barborka 1930; Strahlman 2006; Di Lorenzo et al. 2015; Cherubino Di Lorenzo et al. 2013; Bongiovanni et al. 2021; Di Lorenzo et al. 2019; Lovati et al. 2022; Valente et al. 2022; Tereshko et al. 2023a; Merlino et al. 2023; Caprio et al. 2023).

The objective of this review is two‐fold. First, we summarize migraine pathophysiology from a metabolic standpoint to show that metabolic interventions may be useful. Second, we summarize the relevant literature to show that the KD holds theoretical potential in migraine prophylaxis and could be the target of future, prospective clinical trials.

## 2 Methods

A literature search was conducted on May 6, 2024 using three databases: OVID Medline, Embase, and Cochrane Library. The topic was divided into three concepts: diet, migraine, and efficacy/safety/tolerability. Keywords were generated within each concept to conduct searches.

Keywords for concept one were: KD, diet, ketones, ketosis, ketone bodies, nutrition, food, fasting, glucose, hypergly*, and hypogly*. Keywords for concept two were: migraine, migraine disorders, headache, headache disorders, primary headaches, and cephalalgia. Keywords for concept three were: effective, effectiveness, efficacy, success, successfulness, safe*, taste, tolerability, and palatability. Where possible, depending on the database, keywords were mapped to medical subject headings (MeSH) terms. In an effort to comprehensively analyze the literature, the search criteria were kept broad by using terms such as “headache” rather than only “migraine” or “chronic migraine” to avoid losing any relevant studies. The subsequent manual review of returned articles allowed for only chronic migraine (CM)‐related studies using the KD to be retained for full analysis. The authors specifically did not include KD use in other primary headache disorders since their pathophysiology was not explored herein.

The PICO inclusion criteria were used to screen for relevant articles. The population of interest was adults over 18 years who had a headache or migraine disorder. The intervention of interest was any interventional diet therapy. We allowed uncontrolled and comparator diet populations. The outcomes of interest were reduction in headache days, and also the safety, tolerability, and efficacy of the diet. There were no date cut‐offs.

The initial search returned 25,101 articles (OVID Medline *n* = 574, Embase *n* = 21,781, and Cochrane Library *n* = 2714). Title and abstract screening excluded 25,011 irrelevant articles (OVID Medline *n* = 529, Embase *n* = 21,781, and Cochrane Library *n* = 2728). The excluded studies included pediatric studies (*n* = 47), migraine‐related studies but with no relevance to diet (*n* = 683), and no directly relevant keywords in the title or abstract from concepts 1 to 3 in the search strategy (*n* = 24, 281).

From the remaining 90 articles, 40 duplicates were removed. Full text analysis led to exclusion of six further articles. The six articles that were omitted after full text review were excluded due to: describing history of the KD with limited clinical applicability to critique (*n* = 1), use of the KD in epilepsy rather than migraine (*n* = 2), effects of KD on reproductive hormones rather than migraine (*n* = 1), and electrophysiological effects of the KD with no mention of migraine (*n* = 2).

References of the remaining 44 articles were manually checked to find additional relevant articles. This process led to a further six articles being included, which were not obtained from the original search strategy. The final review thus included 50 articles. Of these 50 articles, 13 were case reports, case series, and observational clinical trials of the KD. The additional 37 articles that were not case studies, case series, or observational studies of the KD in migraine were a mixture of case studies (*n* = 2), a scoping review (*n* = 1), clinical practice guidelines (*n* = 2), and narrative reviewers (*n* = 32). The exact breakdown is as follows: case study about hypoglycemic headache (*n* = 1), case review of cerebral hypoxia causing headache (*n* = 1), scoping review of the metabolic contributions to migraine pathophysiology (*n* = 1), clinical practice guidelines in using the KD for headache disorders (*n* = 2), and narrative reviews of the role of diet in migraine management (*n* = 7); metabolic contributions to migraine pathophysiology (*n* = 13); the effect of KD on sleep (*n* = 1), use of the KD in migraine (*n* = 3), and molecular mechanisms underlying the KD (*n* = 8). The cumulative benefit of including these articles is that they summarize the theoretical reasons why diet‐based interventions may have a place in migraine management—the underlying pathophysiology involves significant metabolic mismatch. This background information also provides context about the difficulty of performing and interpreting real‐world diet studies since they are influenced by the selection of baseline study population, patient weight, glycemic profiles, underlying hormonal variation, chosen interventional diet constituents, diet compliance, monitoring of diet, and so on. This background allows the reader to critically analyze the merits of the included studies as they can see the confounders which may or may not be reported.

## 3 Results and Discussion

### 3.1 Pathophysiology of Migraine in the Various Phases and Its Role in Metabolism

The hypothalamus is likely involved in the early stages of a migraine episode; its activation leads to downstream actions manifesting in energy‐conserving prodromal symptoms up to 48 h before a headache (Gross, Lisicki, et al. 2019). For example, yawning is a means of increasing oxygen levels, food cravings could increase nutritional intake to correct energy shortages, or fatigue and photophobia usually leads to rest which is energy‐conserving. These observations provide interesting insights into a possible link between prodromal symptoms and metabolic dysregulation.

The migraine aura is due to cortical spreading depression; hypoglycemia increases cortical spreading depression while hyperglycaemia protects against its initiation (Gross, Lisicki, et al. 2019). Initiation of cortical spreading depression leads to significant glucose consumption by cells, which further increases the metabolic gap in migraine (Hassan et al. 2020). Once this wave of depolarization activates the trigeminovascular system, it leads to release of neuropeptides such as calcitonin gene‐related peptide (CGRP) and substance P that increases inflammation and can herald a migraine headache. The previously sensitized nerves are vulnerable to external and internal stimuli which can worsen headaches, while the inflammatory milieu sustains the process (Hassan et al. 2020).

During the recovery of cortical spreading depression, further metabolic mismatch occurs due to widespread ion channel activation, high‐oxygen use, and metabolic demand (Wang et al. 2023). The result of all of this is widespread tissue hypoxia, which impairs oxidative phosphorylation in mitochondria, and exacerbates the metabolic disequilibrium (Wang et al. 2023).

### 3.2 Mitochondrial Dysfunction in Migraine

Metabolism is controlled by the cell's mitochondria and its dysfunction is central to migraine (Wang et al. 2023). Mitochondria generate energy via adenosine triphosphate (ATP) through the process of oxidative phosphorylation (Gazerani 2021; Wang et al. 2023; Gross, Lisicki, et al. 2019). Reactive oxygen species (ROS) are a normal biproduct of this process and usually these are sufficiently mopped up by antioxidants (Wang et al. 2023; Gross et al. 2021). In migraine, where antioxidant systems are overwhelmed, ROS formed by cellular respiration persist, leading to release of CGRP which is heavily implicated in migraine pathophysiology (Wang et al. 2023; Gross et al. 2021).

There is substantial evidence that migraine brains are constantly in an energy deficient state when compared to healthy controls (Gazerani 2021; Di Lorenzo et al. 2021; Lai and Niddam 2020; Wang et al. 2023; Gross et al. 2021). The baseline level of energy mismatch, combined with sensory overload to the trigeminovascular system, may be a potential trigger of migraine attacks (Gazerani 2021).

Magnetic resonance spectroscopy studies show that brains of people with migraine have impaired mitochondrial oxidative phosphorylation during and between migraine attacks (Gross, Lisicki, et al. 2019). In fact, ATP levels are 16% lower in migraine brains compared to healthy controls. Also, people with migraine have reduced enzymatic function and substrates needed for mitochondrial oxidative phosphorylation such as NADH hydrogenase, cyclooxygenase, and magnesium. People reporting the most severe migraine had the lowest cerebral ATP concentrations. This may also explain why magnesium is used as a migraine therapeutic, since it is vital for over 300 enzymatic reactions in within the body (Gross, Lisicki, et al. 2019).

All of the above could explain why people with mitochondrial disorders have double the risk of having migraine when compared to the general population (Gross, Lisicki, et al. 2019). People with mitochondrial encephalopathy, lactic acidosis, and stroke‐like‐episodes (MELAS) report a migraine prevalence of 58%, which is over three times higher than the general population prevalence of 18% (Gross, Lisicki, et al. 2019). It may also explain why both migraine and mitochondrial disorders display maternal inheritance. However, not all people with migraine have identifiable mitochondrial genetic mutations so the relationship is complex (Gross, Lisicki, et al. 2019).

### 3.3 Altered Glucose Metabolism

The brain has high‐energy requirements and consumes up to 20% of the body's total oxygen and 25% of the total body glucose for its basal metabolic needs (Del Moro et al. 2022). People with migraine have higher levels of insulin resistance compared to non‐migraine controls, which means that there is less glucose entry into cells to support metabolic functions (Del Moro et al. 2022). While glucose transports in the brain are mostly insulin‐independent, the GLUT‐4 receptor relies on insulin for glucose entry (Del Moro et al. 2022). The GLUT4 receptor is expressed in the cortex, hippocampus, amygdala, and cerebellum which are all areas involved in highly metabolic cognitive functions such as learning (Del Moro et al. 2022). There is suggestion in the literature that peripheral insulin resistance could be accompanied by cerebral insulin resistance, thus worsening the energy deficient state of the migraine brain during periods of hypoglycemia and starvation (Del Moro et al. 2022). The success of corticosteroids for status migrainosus may be, in part, be due to its action in stimulating gluconeogenesis, increasing blood glucose levels, and correcting the protracted energy deficit that caused or prolonged a migraine attack (Gross, Lisicki, et al. 2019).

Natural fasting studies during religious events such as Ramadan (Al‐Hashel et al. 2021) and Yom Kippur (MAaK 1995) show hypoglycemia increases headaches, and can be enough to initiate a migraine in genetically susceptible individuals (Gross, Klement, et al. 2019). The hypometabolic nature of the migraine brain has been demonstrated in FDG‐PET studies comparing migraine brains to healthy controls where both groups were exposed to visual evoked potentials; 90% of migraine brains had visual neuronal activation that exceeded available glucose uptake whereas this only occurred in 15% of normal brains (Gross, Klement, et al. 2019). Any stressor such as hypoglycemia which further stresses this system—that is in a state of deficit at baseline—may possibly worsen migraine. In addition, people with migraine have reduced levels of glucagon and reduced sensitivity to it—thus the body's natural compensation is also impaired in people with migraine, which compounds the hypometabolism (Gross, Lisicki, et al. 2019).

### 3.4 Altered Gut Microbiome

Stepping back from a cellular lens, migraine can be viewed from a body systems perspective. People with migraine have various gastrointestinal symptoms such as nausea, vomiting, and gastroparesis which suggests a vital role for the gastrointestinal system in pathogenesis (Di Lorenzo et al. 2021; Finkel et al. 2013). Migraine is also more common in people with inflammatory bowel disease and coeliac disease, suggesting a shared pathway in pathogenesis (Finkel et al. 2013).

The gut microbiome confers benefits such as immunity, digestion, and metabolic function, but it may also contribute to headaches depending on the microbial constituents (Gazerani 2021). Studies comparing fecal samples of people with migraine to controls have found different compositions of gut bacteria, with increased numbers of Clostridia spp. which are pathogenic, while healthy controls had higher amounts of beneficial bacteria such as *Faecalibacterium*, *Bifidobacterium*, and *Methanobrevibacter* species (Gazerani 2021; Chen et al. 2019; Sgro et al. 2023). Similarly, bacterial species recovered from oral cavities of in people with migraine are significantly different to healthy controls with more bacteria carrying nitrate, nitrite, and nitric oxide reductase genes (Gazerani 2021). However, using probiotics to alter compositions of gut microbiota to a more favorable anti‐inflammatory population for migraine sufferers has not been established (Gazerani 2020, 2021).

### 3.5 Neuroinflammation and Oxidative Stress

Neuroinflammation is central to the pathogenesis of migraine (Gross, Klement, et al. 2019; Sun‐Edelstein and Mauskop 2009; Finelli et al. 2024), and people with both episodic and CM have increased oxidative stress (Di Lorenzo et al. 2021). This may be due to intrinsic factors such as genetic susceptibility or gut microbiome composition. Against this backdrop, there is an unknown trigger activating the hypothalamus with downstream activation of the trigeminal nerve leading to release of pro‐inflammatory cytokines such as CGRP, substance P, nitric oxide (NO), vasoactive intestinal peptide (VIP), and pituitary adenylate cyclase activating peptide (PACAP). All these neurotransmitters are elevated in people with migraine (Finelli et al. 2024; Arzani et al. 2020). This initiates an inflammatory cascade whereby downstream mast cell degranulation, vascular permeability, and local edema sustain the process (Sun‐Edelstein and Mauskop 2009).

### 3.6 Weight Imbalance

Obesity is a risk factor for conversion of episodic migraine to a CM phenotype (Slavin and Ailani 2017). Population studies of over 30,000 people have found that migraine frequency and intensity worsens with increasing body mass index (BMI) (Bigal et al. 2006). This positive association could be due to the inflammatory nature of adipose tissue worsening headaches or the fact that obesity itself increases serum CGRP levels (Bigal et al. 2006). Causation is hard to establish because both migraine and metabolic syndrome co‐occur commonly in modern society (Rainero et al. 2018).

A large systematic review and meta‐analysis of obese people with migraine undergoing bariatric surgery versus lifestyle modifications for weight loss found that weight loss by any means could reduce migraine frequency, intensity, and duration (Vincenzo et al. 2020). These statistically significant results occurred regardless of baseline BMI or extent of weight loss.

To confound our understanding, increased weight predisposes to pathologies such as obstructive sleep apnea and idiopathic intracranial hypertension which cause headaches as a consequence (Martin and Vij 2016). There is also the possibility of obesity and comorbid depression leading to isolating, sedentary behaviors which in turn increases weight and therefore headaches.

In summary, while migraine is a complex and multi‐facet disorder, there is a considerable metabolic component to the disease as reflected in its pathogenesis and clinical features. This had led to manipulation of medications and diet as a means of restoring energy to the hypometabolic brain with variable levels of success, which is explored below. See the graphical abstract for a summary.

### 3.7 The Ketogenic Diet

Ketosis can be induced by two methods; the first is the KD, a low carbohydrate, high‐fat diet with adequate protein intake aimed to replicate the beneficial effects of fasting, while providing a supplemental energy source (Moskatel and Zhang 2022). The second method is by caloric restriction. When a low‐carbohydrate diet is consumed and the person is in an energy‐deficient state, the body rapidly expends available glucose and then uses endogenous fatty acids from adipose tissue for energy (Di Lorenzo et al. 2021). When fatty acids are metabolized in the liver, there is production of the ketone bodies beta hydroxybutyrate (BHB), acetoacetate, and acetone. BHB crosses the blood–brain barrier to provide the brain up to 75% of its energy needs (Di Lorenzo et al. 2021). Acetoacetate may be used in metabolically active tissues for energy; a minority is spontaneously converted to acetone and rapidly excreted via expiration and urination (Di Lorenzo et al. 2021).

There are a variety of types of KD (Di Lorenzo et al. 2021). The “classical” KD is a 4:1 ratio of fats to carbohydrates and proteins meaning 4 g of fat is consumed with a total of 1 g combination of carbohydrate and protein. This classical diet is generally the least tolerable and least palatable due to the high proportion of fats which make up 90% of the daily caloric intake, while proteins and carbohydrates make up 6% and 4%, respectively. Less strict versions of the KD have emerged which all restrict carbohydrates to less than 50 g per day with varying ratios of the other two macronutrients (Barbanti et al. 2017). All KDs aim for physiological ketosis between 0.5–5 mmol/L of serum BHB.

See Table 1 for a summary of the evidence for the KD in CM.

**TABLE 1:** Summary trials of ketogenic diet (KD) in chronic migraine (CM).

Reference	Study type	Population	Intervention	Comparator	Outcome	Timing	Ketosis measure
Schnabel (1928) (USA)	Observational Case series	23 patients with CM (sex and age not recorded).	KD varied from 5:1 to 3:1 fat to carbohydrate ratios. Three immediate start with 5:1 fat to carb; two immediate start 3:1; 18 on gradual start with 3:1 ratio.	Uncontrolled	9/23 patients had headache improvement on KD (but preheadache and postheadache days not consistently recorded).	Follow up range 3–7 months.	Urinary ketones only.
Barborka (1930) (USA)	Observational Case series	50 patients, all female from 16 to 66 years.	KD ratios of carbohydrate, protein, and fat not documented.	Uncontrolled	25/50 had headache resolution, 14/50 had controlled headaches, 11/50 had no benefit. Longest headache freedom post diet was 3 months. No clear relationship between ketosis and headache improvement.	3–36 months follow‐up in various patients based on diet compliance.	Blood ketones.
Strahlman (2006) (USA)	Case report written as letter to editor	One female patient with CM, using KD for weight loss, no age provided.	KD with three to four high protein: low carb shakes per day; fat content not described. Total 200 calories per day.	Uncontrolled	Daily headaches to completely headache free for 14 months of diet compliance.	14 months.	None specified.
Cherubino Di Lorenzo et al. (2013) (Italy)	Observational case report	Two 47‐year‐old twin sisters with CM using KD for weight loss.	KD of < 1 g carbohydrate per day, 1.2–1.8 g protein per day. Fats not specified.	Uncontrolled	Baseline 14–16 MMD reduced to zero MMD while on diet.	3‐month follow‐up.	Urinary ketones checked once daily. No serum ketones.
Di Lorenzo et al. (2015) (Italy)	Randomized control trial	96 patients; all overweight and with CM. All female. Mean age 42 years. Randomized to VLCKD or low calorie SD.	VLCKD for 45 patients for 1 month followed by 5 months of SD; SD for 51 patients for 6 months total. VLCKD included 30 g carb/day, fixed 15 g lipids/day, and normal protein intake. SD was 46% carbs, 24% protein, and 30% total fat per day.	Control group had low calorie SD	In KD group, headache episodes, monthly headache days, and analgesic intake reduced significantly after 1 month of diet; transient worsening when going to SD but better than baseline.	Total 6 months follow‐up in both groups 15 drops out in total during this time.	Urine ketones checked twice per day. No serum ketones.
Di Lorenzo et al. (2019) (Italy)	Randomized, double blind crossover trial	35 overweight patients with CM on KD for weight loss purposes. 29 female; 7 male. Mean age 43 years.	Very low calorie KD (VLCKD) versus very low calorie non‐ketogenic diet (VLCnKD). VLCKD: 30–50 g carb/day, fixed fat 20 g/day, and protein 75 g/day. VLCnKD: 70 g carb/day, fixed fats 20 g/day, and protein 70 g/day.	2 treatment arms, no control arm	6 patients dropped out. 50% responder rate was 26/35 in VLCKD arm versus 3/35 in VLCnKD arm. VLCKD arm had approx. 4 less days of migraine per month.	4 weeks usual diet then 1 month of diet intervention.	Urine ketones. No serum ketones.
Bongiovanni et al. (2021) (Italy)	Observational cohort study	50 patients enrolled and 23 included in statistical analysis. All patients had CM and medication overuse headache. Median age 46 years. 2 male; 36 female.	KD with daily intake of 30 g carbohydrate, 1.5–1.5 g/kg of protein, and variable total caloric intake based on BMI of patient. Lipid intake 35%–80% of total daily calories.	Uncontrolled, single arm	MMD decreased from median 30 to 7.5. Duration of headaches decreased from 24 to 5.5 h. Monthly analgesic use doses dropped from 30 to 6.	3 months of KD, then 1 month of “transition phase” with progressive carbohydrate addition back to diet, and finally 2 months maintenance with low GI diet and 1 free meal per week.	No serum or urine ketones were measured.
Lovati et al. (2022) (Italy)	Observational double arm cohort study	First study: 22 patients with CM (13 in KD arm and 8 to low‐calorie diet/LCD). KD arm: mean age 36; 2 male, 11 female. LCD arm: mean age 50 years, all female. Second study: 31 CM patients (26 in KD arm and 5 on LCD). KD arm 2 male and 22 female. LCD arm 1 male, 5 female but age range not provided for second study.	KD group followed normocaloric or hypocaloric modified Atkins diet. LCD arm had tailored diet with < 40% carbohydrates. No further macronutrient composition breakdown given for either diet.	Control arm was low‐calorie diet	KD arm had less migraine, less intensity of headaches, and less drug intake. No benefit was seen in LCD group.	3 weeks of either diet.	Urine ketones measured daily for 3 weeks. No serum ketones. There was correlation of urine ketones and good response to KD. Higher ketones correlated with less headache frequency.
Valente et al. (2022) (Italy)	Retrospective observational study	23 migraine patients on KD started for migraine prophylaxis. 22 female and 1 male with mean age 47 years.	The exact ratios and composition of the KD was not specified.	Uncontrolled, single arm study	KD arm: patients had less monthly headache days, less analgesic use, and less fat mass. Those who responded versus did not respond to diet had no differences in weight or fat mass loss, that is, efficacy is not just due to weight or fat loss.	Baseline and 3‐month post diet analysis.	Serum ketones were collected in many of the patients but not all, thus not analyzed. No urine ketones.
Tereshko et al. (2023b) (Italy)	Retrospective three arm observational cohort study with prospectively collected data	76 patients with migraine treated with one of 3 variations of KD. 58 females, 18 male; mean age 45 years.	Three different protocols, all with fixed 30 g carbohydrate per day. KD arm 2:1 fat to carb + protein ratio; LGID 1:1 ratio of fats: carb + protein; VLCKD arm varied from 0.43:1 to 0.9:1 fat to carb + protein ratios.	3 treatment arms, no true control	All 3 protocols improved migraine frequency, intensity, MIDAS, and HIT6 scores as well as fatigue levels.	3 months total of diet and then a 3‐month follow‐up.	No serum or urine ketones were measured.
Tereshko et al. (2023a) (Italy)	Retrospective cohort study	60 patients with HFEM and CM randomized to LGID (39) or 2:1 KD (21). 31 female and 8 male in LGID arm with mean age 46 years; 17 female and 4 male in KD arm with mean age 44 years.	2:1 KD versus LGID.	2 treatment arms, no true control	Migraine intensity, frequency, MIDAS, HIT6, fat mass, weight, and BMI improved in both groups.	3‐month follow‐up.	No serum or urine ketones were measured.
Merlino et al. (2023) (Italy)	Observational cohort study of single arm	70 migraine patients on KD for headache prophylaxis. 12 males and 58 females. Mean age 46 years.	1: 1: ketogenic diet with fats equal to total of carb + protein intake. Fixed 30 g carbohydrate per day.	Uncontrolled, single arm	BMI and free fat mass reduced in all on diet. Insomnia decreased from 60% to 40%. All on diet therapy had improved headache frequency, duration, intensity, and MIDAS scores. Sleep changes did not correlate with migraine improvements.	3‐month follow‐up.	No serum or urine ketones were measured.
Caprio et al. (2023) (Italy)	Prospective, single center, randomized, controlled study	57 patients (56 were female) randomized to either VLCKD versus hypocaloric diet. Mean age 42 years.	KD arm 75–105 g protein/day, 30–50 g carb/day, and fixed 20 g/day fats. Hypocaloric arm daily intake was 55% carbs, 30% lipid, and 15% protein.	HBD	Reduction in MMD was higher in VLCKD group than HBD group. Weight loss and quality of life scores were also higher in KD group.	VLCKD is more effective than HBD in reducing MMD in patients with HFEM.	Daily blood ketones. Urine ketones not measured.

Abbreviations: BMI, body mass index; CM, chronic migraine; HBD, hypocaloric balanced diet; HFEM, high frequency episodic migraine; HIT6, Headache Impact Test; KD, ketogenic diet; LCD, low‐calorie diet; LGID, low glycemic index diet; MIDAS, Migraine Disability Assessment; MMD, monthly migraine days; SD, standard diet; VLCKD, very low calorie ketogenic diet; VLCnKD, very low calorie non ketogenic diet.

### 3.8 Proposed Mechanism of Action of the Ketogenic Diet in Migraine

The KD acts on many pathways that were previously discussed including increasing mitochondrial function, reducing inflammation, reducing cortical spreading depression, and normalizing cortical hyperexcitability (Di Lorenzo et al. 2021; Murakami and Tognini 2022; Huynh and Calabrese 2022).

Ketone bodies can supply more ATP than glucose which better counteracts the energy deficit seen in migraine (Di Lorenzo et al. 2021). For example, 100 g of glucose generates 8.7 kg of ATP. However, 100 g of BHB creates 10.5 kg of ATP, while 100 g of acetoacetate can create 9.4 kg of ATP (Di Lorenzo et al. 2021). In addition, the KD has been shown to regenerate and restore function of mitochondrial proteins, increase mitochondrial size and function, increase mitochondrial permeability for more effective oxidative phosphorylation, and remove waste products which would otherwise damage mitochondrial function (Di Lorenzo et al. 2021).

BHB has many functions in reducing inflammation and its downstream effects (Di Lorenzo et al. 2021; Achanta and Rae 2017). It reduces inflammation by directly inhibiting the inflammasome, promoting histone hyperacetylation to promote expression of anti‐inflammatory genes, and as a more effective fuel that glucose, BHB creates less free radicals as a biproduct which also reduces the vicious cycle of inflammation that both triggers and perpetuates migraine (Di Lorenzo et al. 2021).

Ketone bodies may prevent initiation of cortical spreading depression and slow it down if it does occur at all (Di Lorenzo et al. 2021). Ketone bodies increase the inhibitory neurotransmitter GABA while decreasing the neuroexcitatory neurotransmitter glutamate—ultimately reducing migraine onset (Di Lorenzo et al. 2021). In addition, the KD is associated with weight loss and management of obesity could be a further means of achieving migraine control in this population (Di Lorenzo et al. 2021).

### 3.9 Analysis of the Current Evidence for the Ketogenic Diet in Chronic Migraine

Please see Table 1 for a summary of the current evidence in using the KD for CM prevention.

The first reported success of the KD was in 1928 when the author observed that migraine and epilepsy were both disorders of the brain, and that the same KD that he used in epilepsy patients may also improve migraine (Schnabel 1928). He concluded that the diet was helpful in 9 out of 23 patients but each patient was prescribed a different ratio of macronutrients, the speed of transition from a normal diet to a KD varied widely, and there was inconsistent compliance with the diet over 3 months. Despite these shortcomings, the author correctly identified a likely underlying metabolic abnormality in these migraine patients and paved the way for further studies in this area.

A few years later in 1930, a further observational case series of 50 migraine patients placed on the KD was published (Barborka 1930). Half of the patients reported full headache resolution after using the diet for 3 months. This paper did not report the ratio of macronutrients used and speed of transition to the new diet. All 50 patients were reported to be highly‐motivated females which suggests favorable results at least partially due to selection bias. This study correlated headache symptoms with serum ketones. Of the 25 patients who improved, two were always in ketosis and 23 patients were in periodic ketosis. On the other hand, for the 11 patients who had no benefit with the KD, 5 patients never achieved ketosis despite reported full compliance with the diet; two were always in ketosis and three were in periodic ketosis. This study supported the previous observations that metabolic factors likely contribute to migraine and that correcting ketosis alone is unlikely to lead to headache resolution.

No further publications regarding the use of the KD in migraine arose for over 75 years. In 2006 and 2014 respectively, case reports were published of female patients using the KD for weight loss and serendipitously found that their migraine attacks completely disappeared (Strahlman 2006; Cherubino Di Lorenzo et al. 2013). Since these initial uncontrolled observational case series and case reports, prospective cohort studies have been conducted to evaluate the role of the KD in migraine.

In 2015, a randomized control trial of 96 migraine patients was performed, with 45 of them being placed on the KD and 51 being placed on a low‐calorie standard diet (Di Lorenzo et al. 2015). Selection bias was noted due to all trial participants being female. The KD comprised 30 g of carbohydrates, 15 g of lipids, and normal protein intake daily. The comparator diet was a daily intake of 46% carbohydrates, 24% protein, and 30% fat—they did not report grams of each macronutrient used which makes different comparison between the diets difficult. This study showed that both groups had reductions to monthly headache days and acute analgesic use; however, the comparator diet took twice as long to achieve these endpoints. Both groups had urine ketones checked twice per day to ensure compliance with the diet.

In 2021, an uncontrolled observational study of the KD was performed on 50 migraine patients who all also had medication‐overuse‐headache (Bongiovanni et al. 2021). This is a different population to all previous studies. A total of 23 patients completed the study. This diet consisted of fixed 30 g of carbohydrates per day but the lipid intake varied from 35%–80% of the total daily calories. Despite the variation in diet constituents, there were significant reductions to all three of average monthly migraine days, headache duration, and acute analgesic use. In this study, ketones were not checked in urine or blood.

A year later in 2022, a further pilot study was reported involving an observational double‐arm cohort study consisting of 39 migraine patients on the KD and 13 migraine patients on a standard low‐calorie diet (LCD) (Lovati et al. 2022). The KD arm was limited to consuming 10% of total dietary intake in carbohydrates, while the comparator diet included roughly 50% carbohydrate intake per day. In this study, the KD group had less migraine, less intensity of headaches, and less acute drug intake. There was no benefit to any of these parameters in the control arm. Furthermore, there was correlation of levels of ketonuria and headache frequency; higher urine ketones correlated with less headache frequency. Serum ketones were not checked.

Three further studies of the KD were published a year later in 2023 (Tereshko et al. 2023a; Caprio et al. 2023; Tereshko et al. 2023b). The first of these studies looked at three different diet protocols in 76 migraine patients—2:1 KD, low glycaemic index (GI) diet, and very low calorie ketogenic diet (VLCKD). All three protocols led to improved migraine frequency, migraine intensity, MIDAS scores, Headache Impact Test (HIT6) scores, and fatigue levels. However, this study did not measure blood or urine ketones. In a similar vein, a study of 60 patients with CM who were randomized to either the 2:1 KD or a low‐GI diet also found that migraine intensity, migraine frequency, MIDAS scores, HIT6 scores, fat mass, weight, and BMI improved in both groups. This study also did not measure any blood or urine ketones. The third study looked at 57 migraine patients randomly assigned to either a KD of 30–50 g of carbohydrate per day versus a hypocaloric diet, and found that the reduction in migraine days, reduction in body weight, and improvement in quality of life were much greater in the KD arm than the standard therapy arm.

Comparison between studies in Table 1 is difficult due to inconsistencies in methodology. The macronutrient composition was not consistently reported, and where it was, it differed widely both within trial arms of the same study and between studies. Ketones were checked in serum only, urine only, both serum and urine, or neither across different studies. Where they were measured, the timing of samples and threshold for sufficient ketosis was unclear. The speed of transition from a normal diet to a KD was not consistent; we know that this affects palatability of the diet, tolerance, and compliance. Finally, low patient numbers reduce extrapolation of results.

These are, understandably, complexities of real‐world diet studies. Other avoidable confounders included recruitment of only female participants (Di Lorenzo et al. 2015) and investigating the diet in concurrent medication‐overuse‐headache which involves entirely different pathogenetic mechanisms to migraine (Bongiovanni et al. 2021). Despite these limitations, the results seem to show that the KD may reduce migraine days, reduce migraine intensity, and also decrease acute analgesic use. Standardization of patient selection or macronutrient ratio prescriptions in future KD studies in migraine may help guide appropriate patients who would benefit from this intervention.

A few prospective studies have explored pathogenetic reasons for diet success (Di Lorenzo et al. 2019; Valente et al. 2022). The first study observed effects of a LCD with or without ketosis induction; they found that calorie reduction must be accompanied by ketosis to reduce migraine burden (Di Lorenzo et al. 2019). Both groups had similar baseline BMI and glycemic profiles. The second study showed that people achieving ketosis without weight loss still had reduction in headaches (Valente et al. 2022). Finally, though there is evidence that the KD independently improves both migraine and insomnia, there is no direct correlation between better sleep and fewer migraine attacks (Merlino et al. 2023)—thus relationships between ketosis, weight, sleep, and headaches are multidirectional and complex.

International recommendations to guide commencement and maintenance of this diet for migraine are lacking (Cervenka et al. 2021). In 2021 a panel of 12 KD experts was assembled including both neurologists and dietitians from around the world who had managed over 2000 people using this diet (Cervenka et al. 2021). The panel was divided regarding contraindications to the diet, speed of transition from normal diet to KD, baseline blood tests, and monitoring while on the diet. The majority of the panel checked urine and/or blood ketones but there was no consensus about thresholds for sufficient ketosis, timing of testing, or regularity of checks. As such, there is limited scope to recommend this as a migraine preventer without further studies into optimal diet prescription and monitoring.

A dietary intervention to manage migraine allows for a “natural,” drug‐free therapy without side effects of medications which may be appealing to many people. The KD specifically also helps with weight loss and improves insulin sensitivity, in addition to reducing headaches (Batch et al. 2020). It is important, however, that the diet be commenced with dietitian oversight to assess suitability for a dietary intervention in the first instance. Dietetic supervision also avoids transitioning too quickly to this diet from a normal diet which may lead to “keto flu” which involves nausea, gastrointestinal pain, and dizziness (Batch et al. 2020). Dietitian and medical oversight can avoid complications such as renal stones, vitamin deficiencies, hypoproteinemia, muscle cramps, weakness, and hypotension, which may occur if people were to try this diet without expert supervision (Batch et al. 2020).

Once pilot studies have established the diet's safety, tolerability, and efficacy, it is also prudent to monitor for long‐term risks. There is concern that the high‐fat nature of the diet promotes accelerated atherosclerosis which increases risk of cardiovascular disease and stroke (Batch et al. 2020). Rats continuing the KD beyond 22 weeks develop dyslipidemia, hepatic steatosis, and a pro‐inflammatory state (Paoli et al. 2015). Human studies follow participants for maximum of 36 months (Barborka 1930) but did not specifically look at long‐term outcomes.

While real‐world diet studies can vary, we call for future KD studies in migraine populations report (at a minimum) the ratios of macronutrients prescribed (including tolerable ranges), the speed of transition from a normal diet to the KD, whether ketones were checked (including serum vs. urine vs. both) and also the timing of sample collection. Useful additional information would include headache characteristics (baseline and interval changes to monthly headache days, monthly migraine days, headache severity, headache duration, and acute analgesic use), BMI, and patients' glycemic profiles.

### 3.10 Ketogenic Supplements

Given the challenges of long‐term maintenance of the KD, finding less restrictive options with the same therapeutic benefit is of particular interest. Medium chain triglycerides are saturated fatty acids of 6–12 hydrocarbons, which are absorbed directly into the blood stream via the gastrointestinal tract and converted to ketone bodies by the liver (Wolkodoff 2022, Chow et al. 2024). Medium chain triglycerides can be used as part of a KD to increase ketone levels (Wolkodoff 2022, Chow et al. 2024). Clinical trials of medium chain triglyceride supplements, without alteration of dietary intake are limited to two small pilot randomized‐controlled trials (Wolkodoff 2022, Chow et al. 2024). The first reported a reduction in monthly migraine frequency by 53% in the intervention group who received a medium chain triglyceride‐containing micronutrient complex, compared to 6% in the control group who received a placebo supplement (p = 0.0004) after 60 days of supplement use with no adverse events reported in either group (Wolkodoff 2022). The second study reported no difference between the intervention and control groups in monthly migraine frequency (Chow et al. 2024). There was significant early withdrawal rates and adverse events, predominately gastrointestinal, in both the intervention and control groups. Due to low methodological quality of these trials, further research is required to understand the utility of medium chain triglyceride supplements in migraine management.

Oral exogenous ketones in the form of ketone salts and esters have demonstrated to raise blood ketone levels (Soto‐Mota et al. 2019). One study reported that administration of beta‐hydroxybutyrate salt did not confer the same benefits as intensive KD therapy (Putananickal et al. 2022) in people with CM. Therefore, there must be flow‐on metabolic effects of dietary treatment beyond ketosis alone.

## 4 Conclusion

There is emerging literature supporting the KD for CM prevention. Beneficial effects may be driven by correction of cerebral hypometabolism which is central to migraine pathophysiology. Prospective clinical trials are required to evaluate timing, safety, tolerability, efficacy, and putative mechanisms of this interventional diet. We recommend that these future trials report diet macronutrient compositions and their respective percentages of total daily calorie intake, speed of transition to KD, timing, and collection route of ketones (if any). Standardized reporting of KD studies would allow for comparison within and between such studies in the future, which would hopefully contribute to clinical guidelines for CM management.

## Author Contributions

**Lakshini Gunasekera**: conceptualization, writing – original draft, writing – review and editing, methodology, formal analysis. **Jason C. Ray**: conceptualization, writing – review and editing, supervision. **Helmut Butzkueven**: supervision, writing – review and editing. **Terence J. O'brien**: writing – review and editing, supervision. **Elspeth Hutton**: supervision, writing – review and editing. **Neha Kaul**: conceptualization, supervision, writing – review and editing.

## Conflicts of Interest

J.C.R. reports in the last 36 months he has received honoraria for educational presentations for Abbvie, Novartis, and Viatris. He has served on medical advisory boards for Pfizer, Viatris, and Lilly. His institution has received funding for research grants, clinical trials, and projects supported by the International Headache Society, Brain Foundation, Lundbeck, Abbvie, Pfizer, and Aeon. The other authors declare no conflicts of interest.

## Peer Review

The peer review history for this article is available at https://publons.com/publon/10.1002/brb3.70860

## Data Availability

Data sharing not applicable to this article as no datasets were generated or analyzed during the current study.
